# Exact Solution of a Time-Dependent Quantum Harmonic Oscillator with Two Frequency Jumps via the Lewis–Riesenfeld Dynamical Invariant Method

**DOI:** 10.3390/e24121851

**Published:** 2022-12-19

**Authors:** Stanley S. Coelho, Lucas Queiroz, Danilo T. Alves

**Affiliations:** 1 Faculdade de Física, Universidade Federal do Pará, Belém 66075-110, PA, Brazil; 2 Centro de Física, Universidade do Minho, 4710-057 Braga, Portugal

**Keywords:** Lewis–Riesenfeld method, quantum harmonic oscillator, abrupt jumps

## Abstract

Harmonic oscillators with multiple abrupt jumps in their frequencies have been investigated by several authors during the last decades. We investigate the dynamics of a quantum harmonic oscillator with initial frequency $\omega_{0}$, which undergoes a sudden jump to a frequency $\omega_{1}$ and, after a certain time interval, suddenly returns to its initial frequency. Using the Lewis–Riesenfeld method of dynamical invariants, we present expressions for the mean energy value, the mean number of excitations, and the transition probabilities, considering the initial state different from the fundamental. We show that the mean energy of the oscillator, after the jumps, is equal or greater than the one before the jumps, even when $\omega_{1}<\omega_{0}$. We also show that, for particular values of the time interval between the jumps, the oscillator returns to the same initial state.

## 1. Introduction

The quantum harmonic oscillator potential with time-dependent parameters is relevant in modeling several problems in physics, and it has been investigated [1,2,3,4,5,6,7,8,9]. For example, the interaction between a spinless charged quantum particle and a time-dependent external classical electromagnetic field can be studied through a harmonic potential whose frequency depends explicitly on time [4,10,11,12,13], and this is used to model the quantum motion of this particle in a trap [14,15,16,17,18,19]. In the context of quantum electrodynamics, this potential is useful, for instance, to describe the free electromagnetic field in nonstationary media [8,9,20]. In the context of shortcuts to adiabaticity, time-dependent quantum oscillators have also been considered [21,22,23,24,25,26]. Other applications are found in relativistic quantum mechanics, quantum field theory, dynamical Casimir effect, and gravitation [27,28,29,30,31,32,33].

A particular case of a quantum harmonic oscillator with time-dependent parameters that shows sudden frequency jumps is investigated, for instance, in refs. [21,23,24,34,35,36,37,38,39,40,41]. Under such jumps (or any time dependence in the parameters), a classical oscillator in its ground state remains in the same state, whereas a quantum oscillator can become excited [35]. Moreover, the wave functions of quantum harmonic oscillators with time-dependent parameters describe squeezed states [5,9,42], For example, a sudden change in the oscillation frequency of ${}^{85}\mathrm{Rb}$ atoms in the vibrational fundamental state of a one-dimensional optical lattice generates squeezed states [43]. The description of squeezed states is relevant, for instance, in the implementation of schemes for noise minimization in quantum sensors, which increases their sensitivity (see, for instance, ref. [44] and references therein). Subtle points involving the squeezed states for the model of two frequency jumps were investigated, for instance, by Tibaduiza et al. in ref. [40], where the solution for this case was obtained via algebraic method.

In the present paper, we investigate the dynamics of a quantum harmonic oscillator with initial frequency $\omega_{0}$ that undergoes a sudden jump to a frequency $\omega_{1}$ and, after a certain time interval, suddenly returns to its initial frequency. Instead of using the algebraic method used in ref. [40], here, we use the Lewis–Riesenfeld (LR) method of dynamical invariants. The LR method [2,3,4] enables the calculation of the exact wave function of a system subjected, for instance, to a harmonic oscillator potential with time-dependent parameters, such as mass and frequency [5,42,45]. Using this method, we show that the results for the squeeze parameters, the quantum fluctuations of the position and momentum operators, and the probability amplitude of a transition from the fundamental state to an arbitrary energy eigenstate coincide with those found in ref. [40]. In addition, using the same LR method, we also obtain expressions for the mean energy value and for the mean number of excitations (which were not calculated in ref. [40]), as well as for the transition probabilities considering the initial state different from the fundamental (which generalizes the formula found in ref. [40]).

The paper is organized as follows. In Section 2.1, we review some results of the application of the LR method to the quantum harmonic oscillator with time-dependent frequency. In Section 2.2, we define the squeezing parameters and, from these and the oscillator wave function obtained via the LR method, we determine the quantum fluctuations of the position, momentum and Hamiltonian operators, the mean number of excitations, and the transition probabilities between different states. In Section 3, we apply the results of previous sections to the model of ref. [40] and analyze their physical implications. In Section 4, we present our final remarks.

## 2. Analytical Method

### 2.1. The Wave Function of the Harmonic Oscillator via Lewis–Riesenfeld Method

Let us consider the one-dimensional Schrödinger equation for a system whose Hamiltonian $\hat{H}\left(t\right)$ explicitly depends on time [46,47,48],

(1) $$\begin{array}{c} i\hbar \frac{\partial \Psi (x,t)}{\partial t}=\hat{H}\left(t\right)\Psi \left(x,t\right). \end{array}$$

According to the LR method [2,3,4,5,9,42], given an invariant Hermitian operator $\hat{I}\left(t\right)$ which satisfies

(2) $$\begin{array}{c} \frac{\partial \hat{I}\left(t\right)}{\partial t}+\frac{1}{i\hbar }\left[\hat{I}\left(t\right),\hat{H}\left(t\right)\right]=0, \end{array}$$

a particular solution $\Psi_{n}\left(x,t\right)$ of Equation (1) is

(3) $$\begin{array}{c} \Psi_{n}\left(x,t\right)=\exp\left[i\alpha_{n}\left(t\right)\right]\Phi_{n}\left(x,t\right), \end{array}$$

in which $\Phi_{n}\left(x,t\right)$ are the eigenfunctions of $\hat{I}\left(t\right)$, found from

(4) $$\begin{array}{c} \hat{I}\left(t\right)\Phi_{n}\left(x,t\right)=\lambda_{n}\Phi_{n}\left(x,t\right), \end{array}$$

with $\lambda_{n}$ being time-independent eigenvalues of $\hat{I}\left(t\right)$ and $\alpha_{n}\left(t\right)$ phase functions, obtained from the equation

(5) $$\begin{array}{c} \frac{d\alpha_{n}\left(t\right)}{dt}=\int_{-\infty }^{+\infty }dx\;\Phi_{n}^{*}\left(x,t\right)\left[i\frac{\partial }{\partial t}-\frac{1}{\hbar }\hat{H}\left(t\right)\right]\Phi_{n}\left(x,t\right). \end{array}$$

The general solution Ψ(x,t) of Equation (1) is

(6) $$\Psi \left(x,t\right)=\sum_{n=0}^{\infty }C_{n}\Psi_{n}\left(x,t\right),$$

where the time-independent coefficients $C_{n}$ depend only on the initial conditions.

Specifically, for a time-dependent one-dimensional harmonic oscillator with mass $m_{0}$, whose time dependence is contained purely in its oscillation frequency ω(t), the Hamiltonian is given by

(7) $$\begin{array}{c} \hat{H}\left(t\right)=\frac{{\hat{p}}^{2}}{2m_{0}}+\frac{1}{2}m_{0}\;\omega {\left(t\right)}^{2}{\hat{x}}^{2}, \end{array}$$

where $\hat{x}$ and $\hat{p}$ are position and momentum operators, respectively, with $\left[\hat{x},\hat{p}\right]=i\hbar$. An operator $\hat{I}\left(t\right)$ associated with Equation (7) is [4,5,9,42]

(8) $$\begin{array}{c} \hat{I}\left(t\right)=\frac{1}{2}\left\{{\left[\frac{\hat{x}}{\rho (t)}\right]}^{2}+{\left[\rho \left(t\right)\hat{p}-m_{0}\dot{\rho }\left(t\right)\hat{x}\right]}^{2}\right\}, \end{array}$$

wherein ρ(t) is a real parameter which is the solution of the Ermakov–Pinney equation [49,50,51,52]

(9) $$\begin{array}{c} \ddot{\rho }\left(t\right)+\omega {\left(t\right)}^{2}\rho \left(t\right)=\frac{1}{m_{0}^{2}\rho {\left(t\right)}^{3}}. \end{array}$$

The eigenfunctions of $\hat{I}\left(t\right)$, given by Equation (8), are

(10) $$\begin{array}{c} \Phi_{n}\left(x,t\right)=\frac{1}{\sqrt{2^{n}n!}}\Phi_{0}\left(x,t\right)H_{n}\left[\frac{x}{\hbar^{\frac{1}{2}}\rho \left(t\right)}\right], \end{array}$$

where $H_{n}$ are the Hermite polynomials of order *n* [53], $\lambda_{n}=\left(n+1/2\right)\hbar$, and

(11) $$\begin{array}{c} \Phi_{0}\left(x,t\right)={\left[\frac{1}{\pi \hbar \rho {\left(t\right)}^{2}}\right]}^{\frac{1}{4}}\exp\left\{\frac{im_{0}}{2\hbar }\left[\frac{\dot{\rho }\left(t\right)}{\rho (t)}+\frac{i}{m_{0}\rho {\left(t\right)}^{2}}\right]x^{2}\right\}. \end{array}$$

From Equation (5), the functions $\alpha_{n}\left(t\right)$ are given by

(12) $$\begin{array}{c} \alpha_{n}\left(t\right)=-\frac{1}{m_{0}}\left(n+\frac{1}{2}\right)\int_{0}^{t}\frac{dt^{\prime }}{\rho {\left(t^{\prime }\right)}^{2}}. \end{array}$$

Thus, from Equations (3), (10) and (12), the wave function $\Psi_{n}\left(x,t\right)$ associated with the Hamiltonian (7) is

(13) $$\begin{array}{c} \Psi_{n}\left(x,t\right)=\frac{1}{\sqrt{2^{n}n!}}\exp\left[-\frac{i}{m_{0}}\left(n+\frac{1}{2}\right)\int_{0}^{t}\frac{dt^{\prime }}{\rho {\left(t^{\prime }\right)}^{2}}\right]\Phi_{0}\left(x,t\right)H_{n}\left[\frac{x}{\hbar^{\frac{1}{2}}\rho \left(t\right)}\right]. \end{array}$$

For the case in which the frequency is always constant ($\omega \left(t\right)=\omega_{0}$), the solution of Equation (9) is $\rho \left(t\right)=\rho_{0}$, where [4,6,42]

(14) $$\begin{array}{c} \rho_{0}=\frac{1}{\sqrt{m_{0}\omega_{0}}}. \end{array}$$

Therefore, Equation (13) falls back to the wave function of a harmonic oscillator with time-independent mass and frequency, $\Psi_{n}^{\left(0\right)}\left(x,t\right)$, given by [46,47,48]

(15) $$\begin{array}{c} \Psi_{n}^{\left(0\right)}\left(x,t\right)=\frac{1}{\sqrt{2^{n}n!}}{\left(\frac{m_{0}\omega_{0}}{\pi \hbar }\right)}^{\frac{1}{4}}\exp\left[-i\left(n+\frac{1}{2}\right)\omega_{0}t-\frac{m_{0}\omega_{0}x^{2}}{2\hbar }\right]H_{n}\left[{\left(\frac{m_{0}\omega_{0}}{\hbar }\right)}^{\frac{1}{2}}x\right]. \end{array}$$

### 2.2. Squeeze Parameters, Quantum Fluctuations, Mean Number of Excitations, and Transition Probability

As discussed in refs. [5,9,42,54], the quantum states of the time-dependent oscillator, characterized by the wave function $\Psi_{n}\left(x,t\right)$ (Equation (13)), are squeezed. Thus, we can define the squeeze parameter r(t) and the squeeze phase ϕ(t), which specify the squeezed state, in terms of the parameter ρ(t) [55]:

(16) $$\begin{array}{ccc} r(t) & = & \cosh^{-1}\left\{{\left[\frac{m_{0}^{2}\dot{\rho }{\left(t\right)}^{2}+\rho {\left(t\right)}^{-2}+2m_{0}\omega_{0}+m_{0}^{2}\omega_{0}^{2}\rho {\left(t\right)}^{2}}{4m_{0}\omega_{0}}\right]}^{\frac{1}{2}}\right\}, \end{array}$$

(17) $$\begin{array}{ccc} \phi (t) & = & \cos^{-1}\left\{\frac{1+m_{0}\omega_{0}\rho {\left(t\right)}^{2}-2\cosh^{2}\left[r\left(t\right)\right]}{2\sinh[r(t)]\cosh[r(t)]}\right\}, \end{array}$$

with r(t)≥0 and 0≤ϕ(t)≤2π. From Equation (13), one can also obtain the expected value of a given observable $\hat{O}\left(t\right)$ in the state $\Psi_{n}\left(x,t\right)$ as

(18) $$\begin{array}{c} \langle \hat{O}\left(t\right)\rangle \left(n,t\right)=\int_{-\infty }^{+\infty }dx\;\Psi_{n}^{*}\left(x,t\right)\;\hat{O}\left(t\right)\;\Psi_{n}\left(x,t\right), \end{array}$$

which, from Equations (16) and (17), can be written in terms of r(t) and ϕ(t). For the operators $\hat{x}$ and $\hat{p}$, one has [56]:

(19) $$\begin{array}{c} \langle \hat{x}\rangle \left(n,t\right)=\langle \hat{p}\rangle \left(n,t\right)=0, \end{array}$$

(20) $$\begin{array}{c} \langle {\hat{x}}^{2}\rangle \left(n,t\right)=\left(n+\frac{1}{2}\right)\frac{\hbar }{m_{0}\omega_{0}}\{\cosh^{2}\left[r(t)\right]+\sinh^{2}\left[r(t)\right]+2\sinh\left[r(t)\right]\\ \times \cosh\left[r(t)\right]\cos\left[\phi (t)\right]\}, \end{array}$$

(21) $$\begin{array}{c} \langle {\hat{p}}^{2}\rangle \left(n,t\right)=\left(n+\frac{1}{2}\right)m_{0}\omega_{0}\hbar \{\cosh^{2}\left[r(t)\right]+\sinh^{2}\left[r(t)\right]-2\sinh\left[r(t)\right]\\ \times \cosh\left[r(t)\right]\cos\left[\phi (t)\right]\}, \end{array}$$

where it follows, from Equations (7), (20), and (21), that

(22) $$\begin{array}{c} \langle \hat{H}\left(t\right)\rangle \left(n,t\right)=\frac{\langle {\hat{p}}^{2}\rangle \left(n,t\right)}{2m_{0}}+\frac{1}{2}m_{0}\;\omega {\left(t\right)}^{2}\langle {\hat{x}}^{2}\rangle \left(n,t\right). \end{array}$$

From Equations (19)–(21), one finds the variances of the operators $\hat{x}$:

(23) $$\begin{array}{c} \langle {\left[\Delta \hat{x}\right]}^{2}\rangle \left(n,t\right)=\langle {\hat{x}}^{2}\rangle \left(n,t\right)-{\left[\langle \hat{x}\rangle \left(n,t\right)\right]}^{2}, \end{array}$$

and $\hat{p}$:

(24) $$\begin{array}{c} \langle {\left[\Delta \hat{p}\right]}^{2}\rangle \left(n,t\right)=\langle {\hat{p}}^{2}\rangle \left(n,t\right)-{\left[\langle \hat{p}\rangle \left(n,t\right)\right]}^{2}, \end{array}$$

which implies the uncertainty relationship

(25) $$\begin{array}{c} \langle {\left[\Delta \hat{x}\right]}^{2}\rangle \left(n,t\right)\langle {\left[\Delta \hat{p}\right]}^{2}\rangle \left(n,t\right)\geq {\left(n+\frac{1}{2}\right)}^{2}\hbar^{2}\{\cosh^{4}\left[r\left(t\right)\right]+\sinh^{4}\left[r\left(t\right)\right]-2\sinh^{2}\left[r\left(t\right)\right]\\ \times \cosh^{2}\left[r\left(t\right)\right]\cos\left[2\phi \left(t\right)\right]\}. \end{array}$$

Due to the time dependence of the frequency, one can also determine the mean number of excitations $\langle \hat{N}\rangle \left(n,t\right)$ that a system, subjected to this potential, can undergo. This is given by [57,58,59]

(26) $$\begin{array}{c} \langle \hat{N}\rangle \left(n,t\right)=n+\left(2n+1\right)\sinh^{2}\left[r(t)\right]. \end{array}$$

For the fundamental state n=0, one finds $\langle \hat{N}\rangle \left(0,t\right)=\sinh^{2}\left[r(t)\right]$, a result that agrees with refs. [33,56] for vacuum squeezed states. This means that a system, even in the fundamental state, could be excited due to the temporal variations in its frequency. The system subjected to the time-dependent harmonic potential can also make transitions between different states, since time-dependent potentials induce quantum systems to make transitions [46,47,48]. Let us consider that the system is initially at a stationary state $\Psi_{m}^{\left(0\right)}\left(x,t=0\right)$ with frequency $\omega_{0}$ and, due to a modification in its frequency from $\omega_{0}$ to ω(t), it evolves to a new state $\Psi_{m}\left(x,t\right)$ (Equation (13)). In this way, the probability to find the system in the state $\Psi_{n}^{\left(0\right)}\left(x,t\right)$ (Equation (15)), is given by [46,47,48]

(27) $$\begin{array}{c} P{\left(t\right)}_{m\to n}={\left|\int_{-\infty }^{+\infty }dx\;\Psi_{n}^{*(0)}\left(x,t\right)\Psi_{m}\left(x,t\right)\right|}^{2}. \end{array}$$

Using Equations (13) and (15), one can find that $P{\left(t\right)}_{m\to n}=0$ for odd values of |n−m|, and [57,60]

(28) $$\begin{array}{c} P{\left(t\right)}_{m\to n}=\frac{2^{m+n}\min\left(m,n\right){!}^{2}{\left\{\sinh[r(t)]\right\}}^{\left|n-m\right|}}{m!n!\cosh[r(t)]}\\ \times {\left[\sum_{k=\frac{\left|n-m\right|}{2}}^{\frac{n+m}{2}}\frac{\left(\begin{array}{c} \frac{n+m}{2}\\ k \end{array}\right)\left(\begin{array}{c} \frac{n+m+2k-2}{4}\\ \frac{n+m}{2} \end{array}\right)k!}{\left(k-\frac{\left|n-m\right|}{2}\right)!\cosh^{k}\left[r\left(t\right)\right]}\right]}^{2}, \end{array}$$

for even values of |n−m|, where min(m,n) is the smallest value between *m* and *n*. Note that the fact that Equation (28) is nonzero only for even values of |n−m| is related to the parity of the harmonic potential [61]. From Equations (26) (making n=0) and (28), we can relate the probability $P{\left(t\right)}_{m\to n}$ to the mean number of excitations in the fundamental state $\langle \hat{N}\rangle \left(0,t\right)$:

(29) $$\begin{array}{c} P{\left(t\right)}_{m\to n}=\frac{2^{m+n}\min\left(m,n\right){!}^{2}{\left[\langle \hat{N}\rangle \left(0,t\right)\right]}^{\frac{\left|n-m\right|}{2}}}{m!n!{\left[\langle \hat{N}\rangle \left(0,t\right)+1\right]}^{\frac{1}{2}}}\\ \times {\left[\sum_{k=\frac{\left|n-m\right|}{2}}^{\frac{n+m}{2}}\frac{\left(\begin{array}{c} \frac{n+m}{2}\\ k \end{array}\right)\left(\begin{array}{c} \frac{n+m+2k-2}{4}\\ \frac{n+m}{2} \end{array}\right)k!}{\left(k-\frac{\left|n-m\right|}{2}\right)!{\left[\langle \hat{N}\rangle \left(0,t\right)+1\right]}^{\frac{k}{2}}}\right]}^{2}. \end{array}$$

It follows that if the mean number of excitations in the fundamental state is nonzero, then the oscillator will have nonzero probabilities of making transitions between different energy levels. When we consider m=n=0 in Equation (28) (or in Equation (29)), one has the probability of persistence in the fundamental state, and, as a consequence, one can also obtain the probability of excitation, given by $1-P{\left(t\right)}_{0\;\to \;0}$ [40].

## 3. Oscillator with Two Frequency Jumps

Now, we apply the formulas shown in Section 2 to investigate the model discussed in ref. [40], namely an oscillator with

(30) $$\begin{array}{c} \omega \left(t\right)=\left\{\begin{array}{cc} \omega_{0}, & t<0,\\ \omega_{1}, & 0<t<\tau ,\\ \omega_{0}, & t>\tau , \end{array}\right. \end{array}$$

in which $\omega_{0}$ and $\omega_{1}$ are constant frequencies, and τ is the length of the time interval between the frequency jumps.

### 3.1. Solution and General Behavior of the ρ(t) Parameter

Due to the form of Equation (30), the ρ(t) parameter can be written as

(31) $$\begin{array}{c} \rho \left(t\right)=\left\{\begin{array}{cc} \rho_{0}, & t<0,\\ \rho_{1}\left(t\right), & 0<t<\tau ,\\ \rho_{2}\left(t\right), & t>\tau , \end{array}\right. \end{array}$$

where $\rho_{0}$ is given in Equation (14), and $\rho_{1}\left(t\right)$ and $\rho_{2}\left(t\right)$ are calculated next.

#### 3.1.1. Interval 0<t<τ

For the interval 0<t<τ, the equation to be solved is

(32) $$\begin{array}{c} {\ddot{\rho }}_{1}\left(t\right)+\omega_{1}^{2}\rho_{1}\left(t\right)=\frac{1}{m_{0}^{2}\rho_{1}{\left(t\right)}^{3}}, \end{array}$$

with the conditions [6]

(33) $$\begin{array}{c} \rho_{1}\left(t=0\right)=\frac{1}{\sqrt{m_{0}\omega_{0}}},\;\;{\dot{\rho }}_{1}\left(t=0\right)=0. \end{array}$$

The general solution for $\rho_{1}\left(t\right)$ is of the form [50,51]

(34) $$\begin{array}{c} \rho_{1}\left(t\right)={\left[A_{1}\sin^{2}\left(\omega_{1}t\right)+B_{1}\cos^{2}\left(\omega_{1}t\right)+2C_{1}\sin\left(\omega_{1}t\right)\cos\left(\omega_{1}t\right)\right]}^{\frac{1}{2}}, \end{array}$$

and the relationship between the constants $A_{1}$, $B_{1}$, and $C_{1}$ is

(35) $$\begin{array}{c} A_{1}B_{1}-C_{1}^{2}=\frac{1}{m_{0}^{2}\omega_{1}^{2}}. \end{array}$$

Then, applying conditions (33) to Equation (34) and using relation (35), we obtain

(36) $$\begin{array}{c} \rho_{1}\left(t\right)={\left[\frac{\omega_{0}\sin^{2}\left(\omega_{1}t\right)}{m_{0}\omega_{1}^{2}}+\frac{\cos^{2}\left(\omega_{1}t\right)}{m_{0}\omega_{0}}\right]}^{\frac{1}{2}}. \end{array}$$

#### 3.1.2. Interval t>τ

In the interval t>τ, the Ermakov–Pinney equation has the form

(37) $$\begin{array}{c} {\ddot{\rho }}_{2}\left(t\right)+\omega_{0}^{2}\rho_{2}\left(t\right)=\frac{1}{m_{0}^{2}\rho_{2}{\left(t\right)}^{3}}. \end{array}$$

The general solution of Equation (37) is

(38) $$\begin{array}{c} \rho_{2}\left(t\right)={\left[A_{2}\sin^{2}\left(\omega_{0}t\right)+B_{2}\cos^{2}\left(\omega_{0}t\right)+2C_{2}\sin\left(\omega_{0}t\right)\cos\left(\omega_{0}t\right)\right]}^{\frac{1}{2}}, \end{array}$$

with the constants $A_{2}$, $B_{2}$, and $C_{2}$ determined from the relationship

(39) $$\begin{array}{c} A_{2}B_{2}-C_{2}^{2}=\frac{1}{m_{0}^{2}\omega_{0}^{2}}, \end{array}$$

and the conditions for continuity

(40) $$\begin{array}{c} \rho_{1}\left(t=\tau \right)=\rho_{2}\left(t=\tau \right),\;\;\;{\dot{\rho }}_{1}\left(t=\tau \right)={\dot{\rho }}_{2}\left(t=\tau \right). \end{array}$$

Using Equations (36), (38), (39), and (40) results in

(41) $$\begin{array}{c} A_{2}=\frac{1}{m_{0}\omega_{0}^{3}\omega_{1}^{2}}\{\omega_{0}^{2}\omega_{1}^{2}+\left[\left(\omega_{0}^{4}-\omega_{1}^{4}\right)\sin^{2}\left(\omega_{0}\tau \right)-\omega_{0}^{2}\omega_{1}^{2}+\omega_{1}^{4}\right]\sin^{2}\left(\omega_{1}\tau \right)\\ +2\omega_{0}\omega_{1}\left(\omega_{0}-\omega_{1}\right)\left(\omega_{0}+\omega_{1}\right)\sin\left(\omega_{0}\tau \right)\cos\left(\omega_{0}\tau \right)\sin\left(\omega_{1}\tau \right)\cos\left(\omega_{1}\tau \right)\}, \end{array}$$

(42) $$\begin{array}{c} B_{2}=\frac{1}{m_{0}\omega_{0}^{3}\omega_{1}^{2}}\{\omega_{0}^{2}\omega_{1}^{2}+\left[\left(\omega_{1}^{4}-\omega_{0}^{4}\right)\sin^{2}\left(\omega_{0}\tau \right)-\omega_{0}^{2}\omega_{1}^{2}+\omega_{0}^{4}\right]\sin^{2}\left(\omega_{1}\tau \right)\\ -2\omega_{0}\omega_{1}\left(\omega_{0}-\omega_{1}\right)\left(\omega_{0}+\omega_{1}\right)\sin\left(\omega_{0}\tau \right)\cos\left(\omega_{0}\tau \right)\sin\left(\omega_{1}\tau \right)\cos\left(\omega_{1}\tau \right)\}, \end{array}$$

(43) $$\begin{array}{c} C_{2}=\frac{1}{m_{0}\omega_{0}^{3}\omega_{1}^{2}}\{[\left(\omega_{0}^{2}+\omega_{1}^{2}\right)\sin\left(\omega_{0}\tau \right)\cos\left(\omega_{0}\tau \right)\sin\left(\omega_{1}\tau \right)-2\omega_{0}\omega_{1}(\sin^{2}\left(\omega_{0}\tau \right)\\ -1/2)\cos\left(\omega_{1}\tau \right)]\left(\omega_{0}-\omega_{1}\right)\left(\omega_{0}+\omega_{1}\right)\sin\left(\omega_{1}\tau \right)\}. \end{array}$$

#### 3.1.3. General Behavior

**Figure 1:**
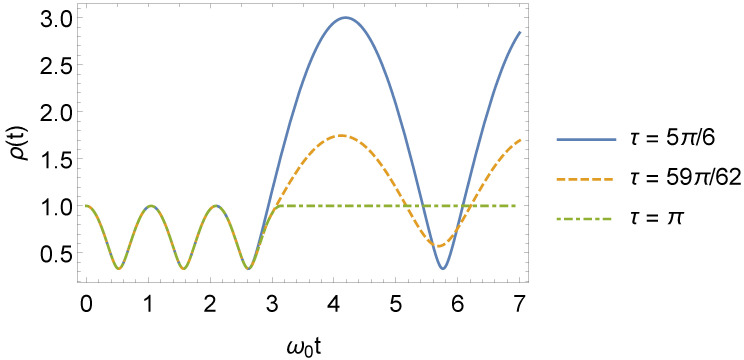
General behavior of ρ(t), as a function of $\omega_{0}t$, with $\omega_{1}=3\omega_{0}$ and different values of τ (we consider, for simplicity, $m_{0}=\omega_{0}=1$ and ℏ=1 in arbitrary units). For $\tau ={\left[\tau_{l+1/2}\right]}_{l=2}=5\pi /6$, we note that the amplitude of oscillation for the ρ(t) parameter is maximum. For τ=59π/62, the amplitude of oscillation is intermediate. Finally, when $\tau ={\left[\tau_{l}\right]}_{l=3}=\pi$, there is no oscillation at all, and it follows that ρ(t) becomes time independent.

The general solution for the ρ(t) parameter is given by Equation (31), with $\rho_{0}$, $\rho_{1}\left(t\right)$ and $\rho_{2}\left(t\right)$ given by Equations (14), (36), (38), (41), (42), and (43). From these equations, it can be seen that the ρ(t) parameter is a periodic function of time. Moreover, even when the frequency returns to its initial value $\omega_{0}$, this parameter will still, in general, be a periodic function of time. However, if we define $\tau_{u}=u\pi /\omega_{1}$ (u>0), and make $\tau =\tau_{l}$, where l∈N, the ρ(t) parameter returns to $\rho_{0}$ (Equation (14)), which means that although the oscillator feels the effect of the change in its frequency when it jumps from $\omega_{0}$ to $\omega_{1}$, if the frequency returns to $\omega_{0}$ at $\tau =\tau_{l}$, for $t>\tau_{l}$, the oscillator behaves as if nothing happened. In other words, if $\tau =\tau_{l}$, the abrupt change in the frequency is imperceptible to the oscillator when $t>\tau_{l}$. On the other hand, when $\tau =\tau_{l+1/2}$, the ρ(t) parameter reaches its maximum value. The behavior of ρ(t) is shown in Figure 1.

### 3.2. Squeeze Parameters

Because the frequency varies abruptly, squeezing occurs in the system [35,36,40]. Thus, now, we calculate the parameters r(t) and ϕ(t) associated with the model in Equation (30). We show that our results for these parameters agree with those found in ref. [40] via an exact algebraic method.

#### 3.2.1. Parameter r(t)

The parameter r(t) for any time interval is given by

(44) $$\begin{array}{c} r\left(t\right)=\left\{\begin{array}{cc} 0, & t<0,\\ r_{1}\left(t\right), & 0<t<\tau ,\\ r_{2}\left(t\right), & t>\tau . \end{array}\right. \end{array}$$

**Figure 2:**
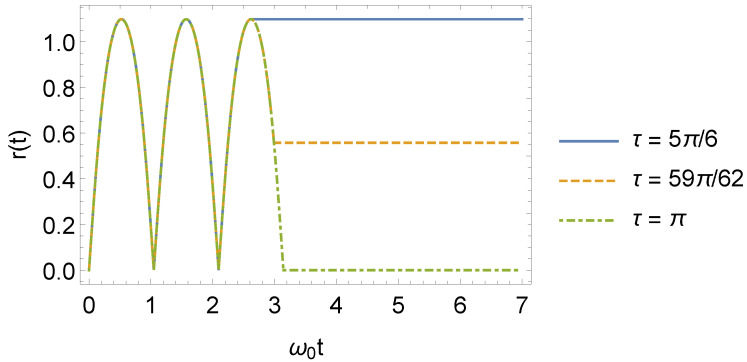
Behavior of the squeeze parameter r(t) as a function of $\omega_{0}t$, where $\omega_{1}=3\omega_{0}$ (we consider $\omega_{0}=1$ in arbitrary units).

Note that r(t<0)=0 because the frequency of the oscillator is time-independent in this interval. Using Equation (36) in Equation (16), we obtain, for the interval 0<t<τ, the squeezing parameter $r_{1}\left(t\right)$, where

(45) $$\begin{array}{c} r_{1}\left(t\right)=\cosh^{-1}\left\{\sqrt{1+{\left(\frac{\omega_{1}^{2}-\omega_{0}^{2}}{2\omega_{0}\omega_{1}}\right)}^{2}\sin^{2}\left(\omega_{1}t\right)}\right\}, \end{array}$$

which is a result that agrees with the one found in ref. [40]. For the interval t>τ, using Equation (38) in Equation (16), we find that $r_{2}\left(t\right)$, with

(46) $$\begin{array}{c} r_{2}\left(t\right)=r_{1}\left(\tau \right), \end{array}$$

this also agrees with ref. [40]. Note that for $\tau =\tau_{l}$, one has $r_{2}\left(t\right)=0$. In Figure 2 (also found in ref. [40]), one can see the behavior of r(t) for some values of τ.

#### 3.2.2. Parameter ϕ(t)

The squeeze phase ϕ(t) for any time interval has the form

(47) $$\begin{array}{c} \phi \left(t\right)=\left\{\begin{array}{cc} \mathrm{undefined}, & t<0,\\ \phi_{1}\left(t\right), & 0<t<\tau ,\\ \phi_{2}\left(t\right), & t>\tau , \end{array}\right. \end{array}$$

where ϕ(t) for t<0 is undefined because there is no squeeze in this interval. Using Equations (36) and (45) in Equation (17), we obtain that the squeeze phase for the interval 0<t<τ, $\phi_{1}\left(t\right)$ is given by

(48) $$\begin{array}{c} \phi_{1}\left(t\right)=\cos^{-1}\left\{\frac{\left(\omega_{0}^{4}-\omega_{1}^{4}\right)\sin^{2}\left(\omega_{1}t\right)}{\sqrt{\left[4\omega_{0}^{2}\omega_{1}^{2}+{\left(\omega_{1}^{2}-\omega_{0}^{2}\right)}^{2}\sin^{2}\left(\omega_{1}t\right)\right]{\left(\omega_{1}^{2}-\omega_{0}^{2}\right)}^{2}\sin^{2}\left(\omega_{1}t\right)}}\right\}, \end{array}$$

which also agrees with ref. [40]. To calculate the squeeze phase in the interval t>τ, the reasoning is analogous, simply substituting Equations (38) and (46) into Equation (17).

**Figure 3:**
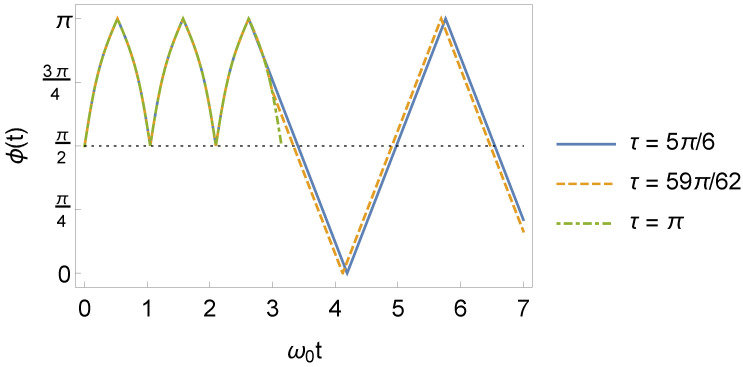
Behavior of the squeeze phase ϕ(t) as a function of $\omega_{0}t$, where $\omega_{1}=3\omega_{0}$ (we consider $\omega_{0}=1$ in arbitrary units).

From Figure 3, it can be seen that the squeezing phase will continue to vary in time, even for t>τ.

Due of this time dependence, the fluctuations of the $\hat{x}$ and $\hat{p}$ operators will continue to depend on time in this interval, as we see later in Section 3.3 (see Equations (23) and (24)). Another point to be observed in Figure 3 concerns the behavior of the squeezing phase in the interval t>τ, when $\tau =\tau_{l}$ (in the specific case of Figure 3, $\tau_{l}=\pi$). Since the squeeze parameter $r_{2}\left(t\right)$ (Equation (46)) is zero in this case, the system is no longer squeezed. Consequently, the squeeze phase is undefined for $\tau_{l}$. Therefore, its effect on the system will be negligible because there will be no more squeezing.

### 3.3. Quantum Fluctuations

The variance of the $\hat{x}$ operator for any time interval is given by

(49) $$\begin{array}{c} \langle {\left[\Delta \hat{x}\right]}^{2}\rangle \left(n,t\right)=\left\{\begin{array}{cc} \langle {\left[\Delta \hat{x}\right]}_{0}^{2}\rangle \left(n\right), & t<0,\\ \langle {\left[\Delta \hat{x}\right]}_{1}^{2}\rangle \left(n,t\right), & 0<t<\tau ,\\ \langle {\left[\Delta \hat{x}\right]}_{2}^{2}\rangle \left(n,t\right), & t>\tau , \end{array}\right. \end{array}$$

where [46]

(50) $$\begin{array}{c} \langle {\left[\Delta \hat{x}\right]}_{0}^{2}\rangle \left(n\right)=\left(n+\frac{1}{2}\right)\frac{\hbar }{m_{0}\omega_{0}}. \end{array}$$

**Figure 4:**
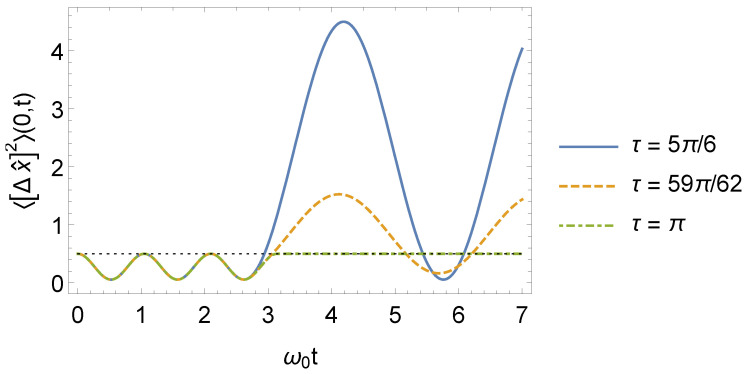
Behavior of the $\langle {\left[\Delta \hat{x}\right]}^{2}\rangle \left(n,t\right)$ for n=0 as a function of $\omega_{0}t$, where $\omega_{1}=3\omega_{0}$ (we consider, for simplicity, $m_{0}=\omega_{0}=1$ and ℏ=1 in arbitrary units).

Substituting Equations (45) and (48) into (23), we find, for the $\hat{x}$ operator in the interval 0<t<τ,

(51) $$\begin{array}{c} \langle {\left[\Delta \hat{x}\right]}_{1}^{2}\rangle \left(n,t\right)=\left[\frac{\omega_{0}^{2}}{\omega_{1}^{2}}\sin^{2}\left(\omega_{1}t\right)+\cos^{2}\left(\omega_{1}t\right)\right]\langle {\left[\Delta \hat{x}\right]}_{0}^{2}\rangle \left(n\right), \end{array}$$

which is in agreement with refs. [35,40]. For the interval t>τ, the procedure is analogous, using Equations (16), (17), and (38) in Equation (23). In Figure 4, we show the behavior of $\langle {\left[\Delta \hat{x}\right]}^{2}\rangle \left(n,t\right)$ for n=0.

Similarly, for the variance of the $\hat{p}$ operator, we have

(52) $$\begin{array}{c} \langle {\left[\Delta \hat{p}\right]}^{2}\rangle \left(n,t\right)=\left\{\begin{array}{cc} \langle {\left[\Delta \hat{p}\right]}_{0}^{2}\rangle \left(n\right), & t<0,\\ \langle {\left[\Delta \hat{p}\right]}_{1}^{2}\rangle \left(n,t\right), & 0<t<\tau ,\\ \langle {\left[\Delta \hat{p}\right]}_{2}^{2}\rangle \left(n,t\right), & t>\tau , \end{array}\right. \end{array}$$

being [46]

(53) $$\begin{array}{c} \langle {\left[\Delta \hat{p}\right]}_{0}^{2}\rangle \left(n\right)=\left(n+\frac{1}{2}\right)\hbar m_{0}\omega_{0}. \end{array}$$

Through Equations (24), (45), and (48), we find, for the interval 0<t<τ, the expression [35,40]

(54) $$\begin{array}{c} \langle {\left[\Delta \hat{p}\right]}_{1}^{2}\rangle \left(n,t\right)=\left[\frac{\omega_{1}^{2}}{\omega_{0}^{2}}\sin^{2}\left(\omega_{1}t\right)+\cos^{2}\left(\omega_{1}t\right)\right]\langle {\left[\Delta \hat{p}\right]}_{0}^{2}\rangle \left(n\right). \end{array}$$

**Figure 5:**
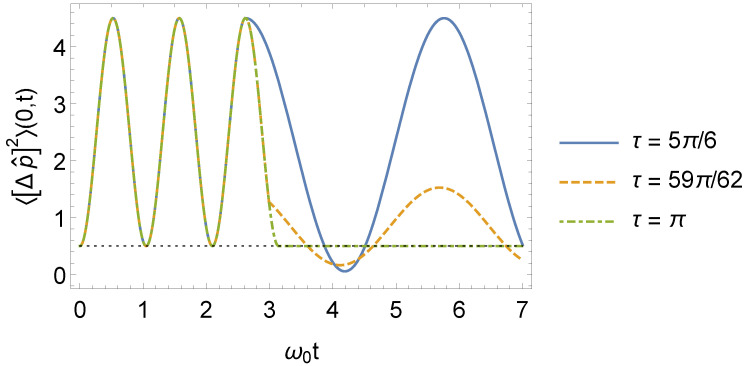
Behavior of the $\langle {\left[\Delta \hat{p}\right]}^{2}\rangle \left(n,t\right)$ for n=0 as a function of $\omega_{0}t$, where $\omega_{1}=3\omega_{0}$ (we consider, for simplicity, $m_{0}=\omega_{0}=1$ and ℏ=1 in arbitrary units).

To calculate the variance of the operator $\hat{p}$ in the interval t>τ, we must use Equations (16), (17), and (38) in Equation (24). The general behavior of $\langle {\left[\Delta \hat{p}\right]}^{2}\rangle \left(n,t\right)$, when n=0, is schematized in Figure 5.

**Figure 6:**
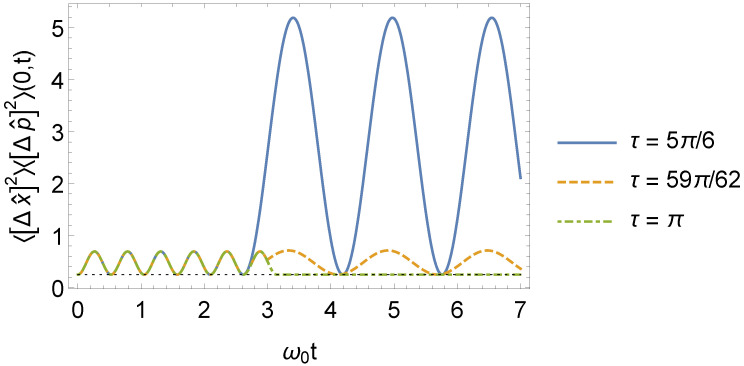
Behavior of the $\langle {\left[\Delta \hat{x}\right]}^{2}\rangle \left(n,t\right)\langle {\left[\Delta \hat{p}\right]}^{2}\rangle \left(n,t\right)$ for n=0 as a function of $\omega_{0}t$, where $\omega_{1}=3\omega_{0}$ (we consider, for simplicity, $m_{0}=\omega_{0}=1$ and ℏ=1 in arbitrary units).

Thus, it is direct to see that the uncertainty relation between these operators in the interval 0<t<τ has the form

(55) $$\begin{array}{c} \langle {\left[\Delta \hat{x}\right]}_{1}^{2}\rangle \left(n,t\right)\langle {\left[\Delta \hat{p}\right]}_{1}^{2}\rangle \left(n,t\right)\geq \langle {\left[\Delta \hat{x}\right]}_{0}^{2}\rangle \left(n\right)\langle {\left[\Delta \hat{p}\right]}_{0}^{2}\rangle \left(n\right)\{1+[\left(\frac{\omega_{1}^{2}-\omega_{0}^{2}}{\omega_{0}\omega_{1}}\right)\sin\left(\omega_{1}t\right)\\ \times \cos\left(\omega_{1}t\right){]}^{2}\}, \end{array}$$

and the uncertainty relation for the interval t>τ is obtained in a similar way. Clearly, when $\omega_{1}=\omega_{0}$, the uncertainty relation (Equation (55)) falls back to the uncertainty relation of a time-independent oscillator [46]. Furthermore, the uncertainty relation for an oscillator with time-independent frequency is also reobtained when $\tau =\tau_{l}$, as shown in Figure 6.

The results found by us (via the LR method) given in Equations (45), (46), (48), (51), and (54) are in agreement with those found in ref. [40]. Hereafter, we use the LR method to obtain new results concerning the model given in Equation (30).

### 3.4. Mean Energy

The expected value of the Hamiltonian operator is identified as the mean energy of the system, that is, $E\left(n,t\right)=\langle \hat{H}\left(t\right)\rangle \left(n,t\right)$. The mean energy for the model in Equation (30) can be written as

(56) $$\begin{array}{c} E\left(n,t\right)=\left\{\begin{array}{cc} E_{0}\left(n\right), & t<0,\\ E_{1}\left(n,t\right), & 0<t<\tau ,\\ E_{2}\left(n,t\right), & t>\tau , \end{array}\right. \end{array}$$

wherein [46]

(57) $$\begin{array}{c} E_{0}\left(n\right)=\left(n+\frac{1}{2}\right)\hbar \omega_{0}. \end{array}$$

For the interval 0<t<τ, through Equations (22), (51), (54), and (57), we find that the mean energy is time-independent, given by

(58) $$\begin{array}{c} E_{1}\left(n,t\right)=\frac{1}{2}\left(1+\frac{\omega_{1}^{2}}{\omega_{0}^{2}}\right)E_{0}\left(n\right). \end{array}$$

When $\omega_{1}=\omega_{0}$, Equation (58) reduces to $E_{1}\left(n,t\right)=E_{0}\left(n\right)$, as expected. Note that for $\omega_{1}/\omega_{0}<1$, $E_{1}\left(n,t\right)<E_{0}\left(n\right)$, whereas for $\omega_{1}/\omega_{0}>1$, we have $E_{1}\left(n,t\right)>E_{0}\left(n\right)$. When $\omega_{1}=0$, which means that the system is free in interval 0<t<τ, we have $E_{1}\left(n,t\right)=E_{0}\left(n\right)/2$. This can also be obtained by making $\omega_{0}\gg \omega_{1}$, which leads to $E_{1}\left(n,t\right)\approx E_{0}\left(n\right)/2$.

For the interval t>τ, from Equations (16), (17), (22), (38), and (57), we have that the mean energy $E_{2}\left(n,t\right)$ is given by

(59) $$\begin{array}{c} E_{2}\left(n,t\right)=\left[1+\frac{1}{2}{\left(\frac{\omega_{1}^{2}-\omega_{0}^{2}}{\omega_{0}\omega_{1}}\right)}^{2}\sin^{2}\left(\omega_{1}\tau \right)\right]E_{0}\left(n\right). \end{array}$$

**Figure 7:**
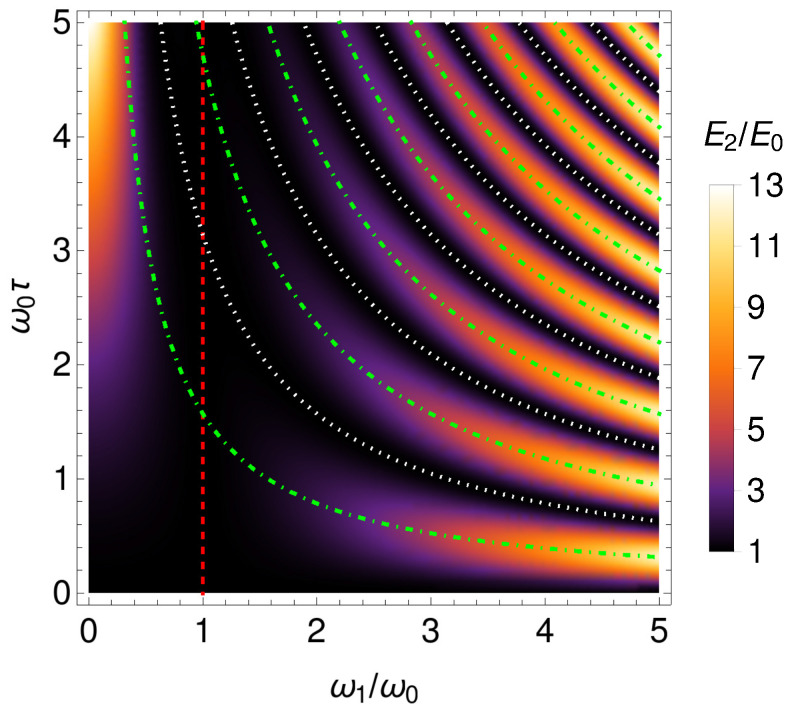
Ratio $E_{2}\left(n,t\right)/E_{0}\left(n\right)$ as a function of $\omega_{0}\tau$ and $\omega_{1}/\omega_{0}$. The dashed line corresponds to $\omega_{1}=\omega_{0}$. The dotted lines correspond to $\tau =\tau_{l}$. The dot–dashed lines correspond to $\tau =\tau_{l+1/2}$.

Note that Equation (59) is independent of *t*, and $E_{2}\left(n,t\right)\geq E_{0}\left(n\right)$, even for $\omega_{1}<\omega_{0}$. The behavior of the ratio $E_{2}\left(n,t\right)/E_{0}\left(n\right)$ is illustrated in Figure 7.

For $\omega_{1}=\omega_{0}$ (dashed line in Figure 7), Equation (59) recovers $E_{2}\left(n,t\right)=E_{0}\left(n\right)$, as expected. Furthermore, for $\tau =\tau_{l}$ (dotted lines in Figure 7), Equation (59) also gives $E_{2}\left(n,t\right)=E_{0}\left(n\right)$. In particular, the energy of this system is maximized when $\tau =\tau_{l+1/2}$ (dot–dashed lines in Figure 7). For $\omega_{1}/\omega_{0}\to 0$, we obtain $E_{2}\left(n,t\right)=\left(1+\omega_{0}^{2}\tau^{2}/2\right)E_{0}\left(n\right)$. Moreover, from Equations (46) and (59), we obtain

(60) $$\begin{array}{c} E_{2}\left(n,t\right)=\left\{2\cosh^{2}\left[r_{2}\left(t\right)\right]-1\right\}E_{0}\left(n\right). \end{array}$$

Thus, while there is squeeze, $E_{2}\left(n,t\right)>E_{0}\left(n\right)$. Therefore, the squeezing caused by the frequency jumps results in an increase in the mean energy of the oscillator with respect to its initial energy $E_{0}\left(n\right)$.

**Figure 8:**
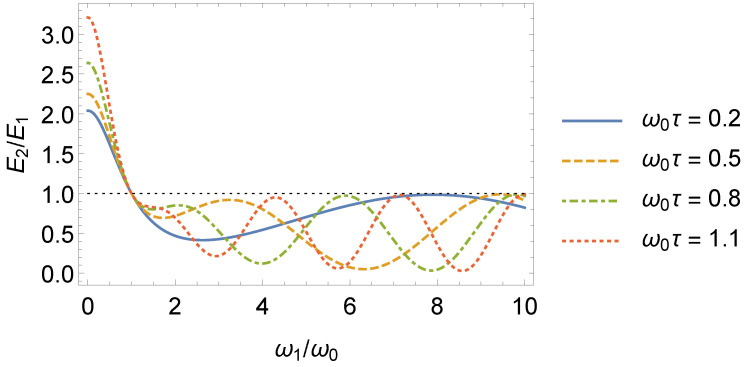
Ratio $E_{2}\left(n,t\right)/E_{1}\left(n,t\right)$ as a function of $\omega_{1}/\omega_{0}$. For $\omega_{1}/\omega_{0}<1$, $E_{2}\left(n,t\right)/E_{1}\left(n,t\right)>1$, whereas for $\omega_{1}/\omega_{0}>1$, $E_{2}\left(n,t\right)/E_{1}\left(n,t\right)\leq 1$.

It is interesting to investigate the behavior of $E_{2}\left(n,t\right)/E_{1}\left(n,t\right)$. Unlike the ratio $E_{2}\left(n,t\right)/E_{0}\left(n\right)$, which is such that $E_{2}\left(n,t\right)/E_{0}\left(n\right)\geq 1$, the ratio $E_{2}\left(n,t\right)/E_{1}\left(n,t\right)$ can be lesser than, equal to, or greater than one, as shown in Figure 8. More specifically, for $\omega_{1}/\omega_{0}<1$, we have $E_{2}\left(n,t\right)/E_{1}\left(n,t\right)>1$, and when $\omega_{1}/\omega_{0}\to 0$, we find $E_{2}\left(n,t\right)=\left(2+\omega_{0}^{2}\tau^{2}\right)E_{1}\left(n,t\right)$. For $\omega_{1}/\omega_{0}>1$, the ratio $E_{2}\left(n,t\right)/E_{1}\left(n,t\right)$ oscillates between zero and one (see Figure 8). We highlight that, besides the trivial case $\omega_{1}=\omega_{0}$, there are other values of the ratio $\omega_{1}/\omega_{0}$ that result in $E_{2}\left(n,t\right)=E_{1}\left(n,t\right)$.

### 3.5. Mean Number of Excitations

The mean number of excitations, $\langle \hat{N}\rangle \left(n,t\right)$, for the model in Equation (30), is given by

(61) $$\begin{array}{c} \langle \hat{N}\rangle \left(n,t\right)=\left\{\begin{array}{cc} {\langle \hat{N}\rangle }_{0}\left(n\right), & t<0,\\ {\langle \hat{N}\rangle }_{1}\left(n,t\right), & 0<t<\tau ,\\ {\langle \hat{N}\rangle }_{2}\left(n,t\right), & t>\tau , \end{array}\right. \end{array}$$

where [46]

(62) $$\begin{array}{c} {\langle \hat{N}\rangle }_{0}\left(n\right)=n. \end{array}$$

Given this, for the interval 0<t<τ, by means of Equations (26) and (45), we have

(63) $$\begin{array}{c} {\langle \hat{N}\rangle }_{1}\left(n,t\right)=n+\left(n+\frac{1}{2}\right)\left[\frac{1}{2}{\left(\frac{\omega_{1}^{2}-\omega_{0}^{2}}{\omega_{0}\omega_{1}}\right)}^{2}\sin^{2}\left(\omega_{1}t\right)\right]. \end{array}$$

We remark the time dependence in ${\langle \hat{N}\rangle }_{1}\left(n,t\right)$, whereas the mean energy in the interval 0<t<τ (Equation (58)) is time-independent.

For the interval t>τ, through Equations (26) and (46), we obtain ${\langle \hat{N}\rangle }_{2}\left(n,t\right)$, given by

(64) $$\begin{array}{c} {\langle \hat{N}\rangle }_{2}\left(n,t\right)={\langle \hat{N}\rangle }_{1}\left(n,\tau \right). \end{array}$$

**Figure 9:**
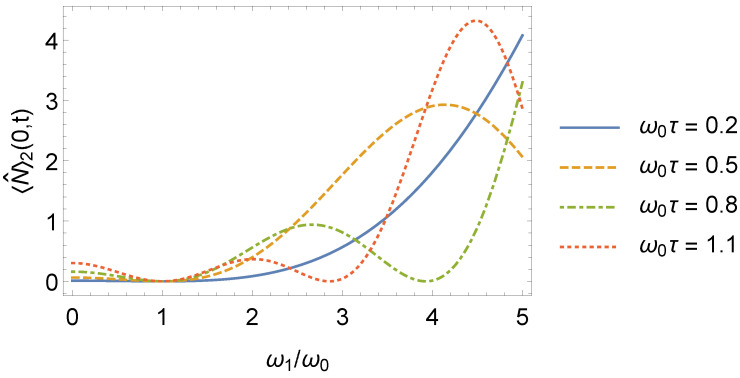
Some examples of mean number of excitations ${\langle \hat{N}\rangle }_{2}\left(0,t\right)$ that an oscillator could undergo as a function of $\omega_{1}/\omega_{0}$ for different values of $\omega_{0}\tau$.

We also remark that for $\tau =\tau_{l}$, the behavior of the system returns to that of the time-independent oscillator found before the frequency jumps, i.e, ${\langle \hat{N}\rangle }_{2}\left(n,t\right)={\langle \hat{N}\rangle }_{0}\left(n\right)$. We highlight that there is excitation even for n=0, which means that, under jumps in its frequency, a quantum oscillator initially in its ground state can become excited (a classical oscillator in its ground state would remain in the same state). In addition, excitation can also occur when $\omega_{1}/\omega_{0}<1$, as shown in Figure 9.

**Figure 10:**
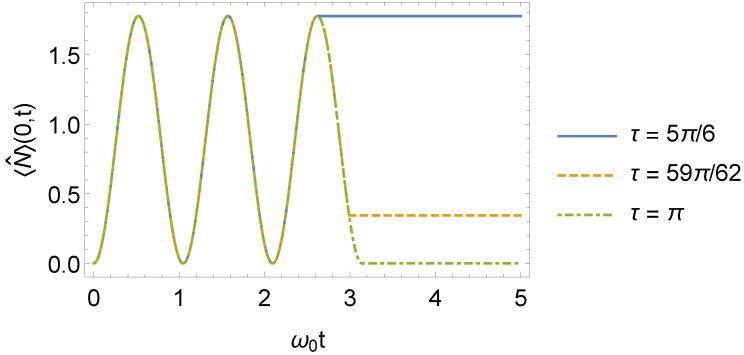
Mean number of excitations $\langle \hat{N}\rangle \left(0,t\right)$ as a function of $\omega_{0}t$ for different values of τ, where $\omega_{1}=3\omega_{0}$ (we consider $\omega_{0}=1$ in arbitrary units).

Using Equations (59) and (64), we can also write $E_{2}\left(n,t\right)$ as

(65) $$\begin{array}{c} E_{2}\left(n,t\right)=\left[{\langle \hat{N}\rangle }_{2}\left(n,t\right)+\frac{1}{2}\right]\hbar \omega_{0}, \end{array}$$

which has the same structure as the expression for the energy eigenvalues of a time-independent oscillator (see Equation (57)). The time evolution of $\langle \hat{N}\rangle \left(0,t\right)$, given by Equation (61) for n=0, is shown in Figure 10.

### 3.6. Transition Probability

The general transition probability, $P{\left(t\right)}_{m\to n}$, for the model in Equation (30), is given by

(66) $$\begin{array}{c} P{\left(t\right)}_{m\to n}=\left\{\begin{array}{cc} \delta_{m,n}, & t<0,\\ P_{1}{\left(t\right)}_{m\to n}, & 0<t<\tau ,\\ P_{2}{\left(t\right)}_{m\to n}, & t>\tau , \end{array}\right. \end{array}$$

with $\delta_{m,n}$ being the Kronecker delta. Using Equations (28) and (45) (or Equations (29) and (63) with n=0), we find, for the interval 0<t<τ, $P_{1}{\left(t\right)}_{m\to n}$, whose expression is

(67) $$\begin{array}{c} P_{1}{\left(t\right)}_{m\to n}=\frac{2^{m+n}\min\left(m,n\right){!}^{2}{\left[\left(\frac{\omega_{1}^{2}-\omega_{0}^{2}}{2\omega_{0}\omega_{1}}\right)\sin\left(\omega_{1}t\right)\right]}^{\left|n-m\right|}}{m!n!{\left[1+{\left(\frac{\omega_{1}^{2}-\omega_{0}^{2}}{2\omega_{0}\omega_{1}}\right)}^{2}\sin^{2}\left(\omega_{1}t\right)\right]}^{\frac{1}{2}}}\\ \times {\left[\sum_{k=\frac{\left|n-m\right|}{2}}^{\frac{n+m}{2}}\frac{\left(\begin{array}{c} \frac{n+m}{2}\\ k \end{array}\right)\left(\begin{array}{c} \frac{n+m+2k-2}{4}\\ \frac{n+m}{2} \end{array}\right)k!}{\left(k-\frac{\left|n-m\right|}{2}\right)!{\left[1+{\left(\frac{\omega_{1}^{2}-\omega_{0}^{2}}{2\omega_{0}\omega_{1}}\right)}^{2}\sin^{2}\left(\omega_{1}t\right)\right]}^{\frac{k}{2}}}\right]}^{2}, \end{array}$$

for even values of |n−m|, and $P{\left(t\right)}_{m\to n}=0$ for odd values of |n−m|. Since the $r_{1}\left(t\right)$ parameter (Equation (45)) in this interval is explicitly time-dependent, consequently, the transition probability m→n will also depend.

For the interval t>τ, using Equation (46) in Equation (28) (or Equations (29) and (64) with n=0), we see that the result is

(68) $$\begin{array}{c} P_{2}{\left(t\right)}_{m\to n}=P_{1}{\left(\tau \right)}_{m\to n}. \end{array}$$

**Figure 11:**
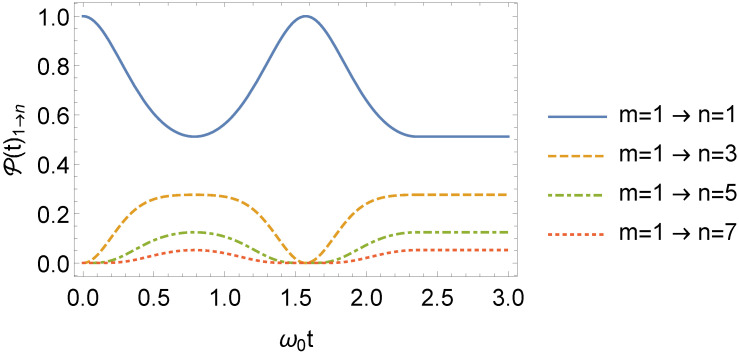
Behavior of $P{\left(t\right)}_{1\to n}$, as a function of $\omega_{0}t$, with $\omega_{1}=2\omega_{0}$ and $\tau =3\tau_{1}/2=3\pi /4$ (we consider $\omega_{0}=1$ in arbitrary units).

**Figure 12:**
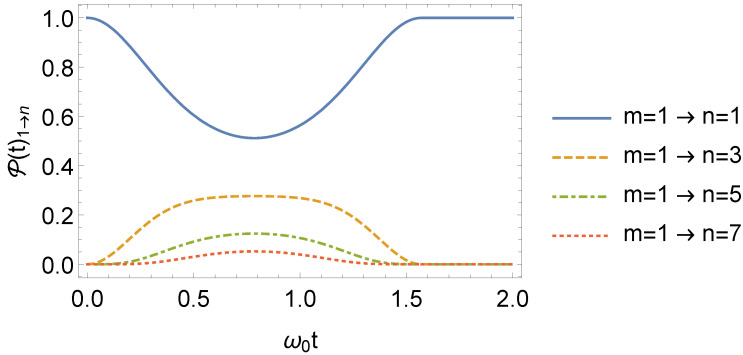
Behavior of $P{\left(t\right)}_{1\to n}$, as a function of $\omega_{0}t$, with $\omega_{1}=2\omega_{0}$ and $\tau =\tau_{1}=\pi /2$ (we consider $\omega_{0}=1$ in arbitrary units).

Thus, even when the frequency returns to $\omega_{0}$ after an instant τ, one can find a nonzero m→n transition probability, depending on the value of τ. It is noticeable from Equation (67) that $P{\left(t\right)}_{m\to n}=P{\left(t\right)}_{n\to m}$, with such symmetry being a consequence of the parity of the potential in Equation (7) [61]. The behavior of $P{\left(t\right)}_{1\to n}$ is illustrated in Figure 11 (for $\tau =3\tau_{1}/2$) and Figure 12 (for $\tau =\tau_{1}$). In Figure 11, as *n* increases, $P{\left(t\right)}_{1\to n}$ decreases. We highlight that for $t>\tau =\tau_{1}$ in Figure 12, $P{\left(t\right)}_{1\to 1}=1$ or, in other words, the oscillator remains in its same initial state.

In addition, for m=0, Equation (68) gives

(69) $$\begin{array}{c} P_{2}{\left(t\right)}_{0\;\to \;n}=\frac{n!{\left(\frac{\omega_{1}^{2}-\omega_{0}^{2}}{2\omega_{0}\omega_{1}}\right)}^{n}\sin^{n}\left(\omega_{1}\tau \right)}{2^{n}{\left(\frac{n}{2}!\right)}^{2}{\left[1+{\left(\frac{\omega_{1}^{2}-\omega_{0}^{2}}{2\omega_{0}\omega_{1}}\right)}^{2}\sin^{2}\left(\omega_{1}\tau \right)\right]}^{\frac{n+1}{2}}}, \end{array}$$

where n=0,2,4,…, recovering one of the results found in ref. [40]. Thus, Equation (68) generalizes the result for the transition probability found in ref. [40]. By also making n=0 in Equation (69), we find the probability of the oscillator of persisting in the fundamental state, and the probability of the oscillator being excited, after the frequency returns to $\omega_{0}$, which is given by $1-P_{2}{\left(t\right)}_{0\;\to \;0}$.

## 4. Final Remarks

Using the Lewis–Riesenfeld method, we investigated the dynamics of a quantum harmonic oscillator that undergoes two abrupt jumps in its frequency (Equation (30)). We reobtained the analytical formulas of ref. [40] for the squeeze parameters (Equations (45), (46), and (48)), the quantum fluctuations of the position (Equation (51)) and momentum (Equation (54)) operators, and the probability amplitude of a transition from the fundamental state to an arbitrary energy eigenstate (Equation (69)). We also obtained expressions for the mean energy value (Equations (58) and (59)), the mean number of excitations (Equations (63) and (64)) (which were not calculated in ref. [40]), and for the transition probabilities considering the initial state different from the fundamental (Equations (67) and (68)) (which generalizes the formula found in ref. [40]).

We found that, as expected, the mean energy of the system is independent of time in each one of the intervals: t<0, 0<t<τ, and t>τ. Moreover, we showed that the mean energy of the oscillator after the jumps is equal or greater than that before these jumps, even when $\omega_{1}<\omega_{0}$. We also obtained, for $t>\tau \neq \tau_{l}$, a non-null value for the mean number of excitations when the oscillator starts in the fundamental state (Equations (58) and (59) with n=0), which means that, under the jumps in its frequency, a quantum oscillator, initially in the ground state, can become excited. We showed that transitions between arbitrary *m* and *n* states only occur if |n−m| is an even number. We highlighted that, for $t>\tau \neq \tau_{l}$ and a fixed value of *m*, as *n* increases, $P{\left(t\right)}_{m\to n}$ decreases. Finally, we showed that, for $t>\tau =\tau_{l}$, $P{\left(t\right)}_{m\to n}=\delta_{m,n}$, so that the oscillator returns to the same initial state (this generalizes, for any initial state *m*, the result found in ref. [40] for m=0).

## Data Availability

Not applicable.
